# Photothermal and radiotherapy with alginate-coated gold nanoparticles for breast cancer treatment

**DOI:** 10.1038/s41598-024-60396-w

**Published:** 2024-06-10

**Authors:** Mohammadreza Ghaffarlou, Hamid Rashidzadeh, Ali Mohammadi, Navid Mousazadeh, Murat Barsbay, Ali Sharafi, Mahmoud Gharbavi, Hossein Danafar, Siamak Javani

**Affiliations:** 1 Department of Chemistry, Hacettepe University, 06800 Beytepe, Ankara, Turkey; 2 Zanjan Pharmaceutical Biotechnology Research Center, Zanjan University of Medical Sciences, Zanjan, Iran; 3 Nanotechnology Research Center, Medical Basic Sciences Research Institute, Ahvaz Jundishapur University of Medical Sciences, Ahvaz, Iran; 4 Pain Research Center, Imam Khomeini Hospital Clinical Research Development Unit, Ahvaz Jundishapur University of Medical Sciences, Ahvaz, Iran; 5 Medical Cellular and Molecular Research Center, Golestan University of Medical Sciences, Gorgan, Iran; 6 School of Advanced Technologies in Medicine, Golestan University of Medical Sciences, Gorgan, Iran

**Keywords:** Radiotherapy, Radiosensitizer, Radiation, Photothermal, Gold nanoparticle, Green synthesis, Cancer, Nanoscience and technology

## Abstract

Radiation therapy and phototherapy are commonly used cancer treatments that offer advantages such as a low risk of adverse effects and the ability to target cancer cells while sparing healthy tissue. A promising strategy for cancer treatment involves using nanoparticles (NPs) in combination with radiation and photothermal therapy to target cancer cells and improve treatment efficacy. The synthesis of gold NPs (AuNPs) for use in biomedical applications has traditionally involved toxic reducing agents. Here we harnessed dopamine (DA)-conjugated alginate (Alg) for the facile and green synthesis of Au NPs (Au@Alg-DA NPs). Alg-DA conjugate reduced Au ions, simultaneously stabilized the resulting AuNPs, and prevented aggregation, resulting in particles with a narrow size distribution and improved stability. Injectable Au@Alg-DA NPs significantly promoted ROS generation in 4T1 breast cancer cells when exposed to X-rays. In addition, their administration raised the temperature under a light excitation of 808 nm, thus helping to destroy cancer cells more effectively. Importantly, no substantial cytotoxicity was detected in our Au@Alg-DA NPs. Taken together, our work provides a promising route to obtain an injectable combined radio enhancer and photothermally active nanosystem for further potential clinic translation.

## Introduction

Radiation therapy or radiotherapy is a commonly utilized therapeutic approach in the treatment of various forms of cancer^1–3^. This treatment involves the application of high doses of ionizing radiation to the region of the body affected by the cancer. The benefits of radiotherapy are numerous, including a low risk of adverse effects, relatively low cost compared to other cancer treatments, and the ability to accurately target and destroy cancer cells while sparing healthy tissue. In addition, it may be used in combination with other therapies such as chemotherapy^4,5^ and phototherapy^1,6^ in order to improve the overall efficacy of the treatment plan. However, it should be noted that there are also certain drawbacks associated with radiation therapy. Some patients may experience side effects such as skin irritation, fatigue and nausea. In some cases, radiation therapy may also damage healthy tissue, leading to long-term consequences such as infertility or an increased risk of secondary malignancies^7,8^.

Photothermal therapy, another cancer treatment approach, utilizes laser light to generate heat, thereby thermally destroying cancer cells. This therapy offers patients several advantages, including the ability to precisely target and destroy cancer cells, while minimizing damage to healthy tissue, making it a minimally invasive approach^9^. Despite its benefits, photothermal therapy is associated with some drawbacks, such as the need for expensive equipment not be readily available in all medical facilities, and the possibility of inadequate efficacy against certain forms of cancer, as well as adverse effects at the treatment site. Additionally, as a relatively new therapeutic modality, further research is needed to fully understand its benefits and limitations^10–12^.

Combination therapies that integrate nanoparticles (NPs) with radiation and photothermal treatment represent a novel and potentially fruitful strategy for fighting cancer. NPs may be designed to selectively target cancer cells and deliver therapeutic agents such as drugs or contrast agents to improve the efficacy of treatment. This can be accomplished through a combination of engineering and biological processes. NPs, when used in combination with radiotherapy, have the potential to boost both the administration of radiation to the target region as well as the efficiency of the treatment. NPs may be engineered to absorb light and convert it into heat as part of a treatment called photothermal therapy. This raises the temperature at the location of the tumor, which helps cancer cells be destroyed more effectively^13–15^. Combinational therapy approaches hold promise for surpassing conventional treatments. They offer the potential for more precise targeting of cancer cells and a greater success rate due to the enhanced effectiveness of radiosensitizing agents. A radiosensitizer agent is a substance with a high-Z element that boosts the efficacy of radiation treatment. Radiosensitizer agents act by enhancing the sensitivity of cancer cells to radiation, making them more vulnerable to radiation therapy-induced damage^16–18^. However, there is still a long way to go before combination therapies based on NPs are implemented and optimized to their full potential in further studies to enable researchers to design both safe and effective^19,20^.

Gold NPs (AuNPs) can be synthesized using chemical reducing agents such as sodium borohydride and hydrazine to reduce gold ions into zero-valent AuNPs^21,22^. This method is commonly used in laboratory settings, but it has some drawbacks due to the toxicity of these agents that pose a biological risk. Recently, green methods have emerged for synthesizing NPs using natural polymers and safe organic compounds such as chitosan, starch, polypeptides, heparin, and hyaluronic acid. Some of these compounds can both produce and simultaneously stabilize metallic NPs, enabling a green one-step synthesis process^23,24^. As a natural and biocompatible polymer, alginate (Alg) offers a green route for the synthesis of AuNPs without the use of toxic reducing agents^23,25^. However, reduction of gold ions to AuNPs using Alg is often not efficient and can result in the formation of particles with a wide size distribution. Here, in order to overcome this obstacle, conjugation of dopamine (DA) with Alg has been proposed. DA is a natural biocompatible molecule containing catechol groups that can be oxidized to reduce Au ions to form AuNPs. Alg-DA conjugate yielded monodisperse and stable AuNPs in a green and one-pot route. Moreover, the use of Alg-DA has the potential to offer a safer, more environmentally friendly and more efficient AuNPs synthesis method compared to chemical reduction, in addition to its enhanced application potential in biomedicine. The resulting Au@Alg-DA NPs were applied as biocompatible and injectable radioenhancers to improve the efficacy of radiotherapy and photothermal therapy.

## Materials and methods

All methods used in this study were conducted in accordance with relevant guidelines and regulations of the Ethics Committee of the Ahvaz Jundishapur University of Medical (Code: 99026551).

### Materials

Sodium alginate was purchased from ISOLAB (Turkey). Dopamine, EDC, NHS, HAuCl_4_·3H_2_O, Calcein-AM, Propidium iodide (PI), 2ʹ,7ʹ-Dichlorofluorescin Diacetate (DCFH-DA) were purchased from Sigma Aldrich Inc (St. Louis, Missouri, United States).

### Methods

All methods used in this study were conducted in accordance with relevant guidelines and regulations of the Ethics Committee of the Ahvaz Jundishapur University of Medical. The experimental protocols were approved by the Ethics Committee of the Ahvaz Jundishapur University of Medical.

#### Synthesis of dopamine-modified alginate (Alg-DA)

The literature-reported synthesis protocol was followed to prepare Alg-DA^26,27^. Briefly, 100 mL of deionized water was used to dissolve 1.0 g of Alg, then 986.0 mg of EDC and 582.0 mg of NHS were added to the solution. To completely activate the carboxylic acid groups on Alg, the reaction mixture was stirred at room temperature for 30 min before being combined with 1.92 g of DA. After the addition of DA, the reaction mixture was stirred at room temperature for 24 h. To prevent self-polymerization of DA during the process, the reaction was carried out in the absence of oxygen by degassing the solution by N_2_.The synthesized Alg-DA was precipitated three times with ethanol before being lyophilized. The synthesized Alg-DA was characterized by Ultraviolet–Visible (Uv–Vis) and proton nuclear magnetic resonance (^1^H NMR) spectroscopy.

#### Synthesis of Au@Alg-DA NPs

Under vigorous stirring, 40.0 mg of Alg-DA was dissolved in 100 mL of boiling deionized water, followed by addition of 14.2 µL of aqueous tetra-chloroauric acid solution (HAuCl_4_, 1.0 M). The mixture was stirred continuously at 120 °C for 2 h. The color of the reaction mixture changed to ruby red, suggesting the formation of metallic Au NPs. Au@Alg-DA NPs were purified via dialysis against deionized water.

#### Synthesis of Au@Alg NPs

To see the effect of DP on Au NPs formation, nanoparticle synthesis was repeated with neat Alg. 20.0 mg of Alg was added to 50 mL of boiling deionized water under vigorous stirring. Subsequently, 7.6 µL of 1.0 M HAuCl_4_ was rapidly added to the solution and the mixture was stirred continuously for 2 h at 120 °C. The synthesized Au@Alg NPs were purified via dialysis process.

### Characterization

The structure of Alg-DA was analyzed by ^1^H NMR using a 400 MHz NMR spectrometer (Bruker; Germany). Uv–Vis spectrometery (Perkin Elmer, lambda 25; United States) was used to optical examination of the samples. Transmission electron microscopy (TEM, FEI 120 kV; United States), and field emission scanning electron microscopy (FESEM, FEI Quanta 200FEG; United States) were used to study the morphology and size of the NPs. Dynamic light scattering (DLS; Malvern Instruments, Worcestershire, UK, model Nano ZS90) was used to determine the hydrodynamic diameter and surface charge (ζ) of the particles. The crystalline structure of the samples was characterized by X-ray diffraction (XRD, Malvern Panalytical Empyrean; Netherlands) by using Cu-Kα1 radiation source, λ = 0.15406 nm. XRD data at 2θ ranging from 5° to 85° were collected with a scan step size of 0.02°.

### Photothermal effects of Au@Alg-DA

For measurement of temperature change mediated by Au@Alg-DA NPs, light emitting diodes (LED) illumination (1 W/cm^2^) at 808 nm was delivered through a plate containing the NPs (500 µg/mL). PBS solution was used as a control. Using a digital thermometer, the temperature of the solution was monitored.

### In vitro experiment

To ensure reproducibility and quality control, well-characterized cell lines were obtained commercially from the Pasteur Institute of Iran.

#### MTT assay

The 3-(4,5-dimethylthiazol-2-yl)-2,5-diphenyltetrazolium bromide (MTT) assay is a commonly used method to determine the viability of cells in vitro. The basic principle of the assay involves adding the MTT reagent and allowing it to be converted to formazan by the mitochondria of viable cells. After incubation, the culture medium is removed and replaced with DMSO to dissolve the formazan, which is then quantified by measuring its absorbance at 570/640 nm using a microplate reader. The absorbance value is proportional to the number of viable cells in the well and cell viability is expressed as a percentage of untreated control cells. The MTT assay is a fast, simple, and reliable method for evaluating cell viability and is widely used in cell biology research. It is important to note that the cytotoxicity of the prepared samples was determined by performing an MTT assay on normal human endothelial cells (HUVECs) to evaluate the biocompatibility on normal cells at concentrations of 18.75 μg/mL, 37.5 μg/mL, 75 μg/mL, and 300 μg/mL. After the treatment period was completed, MTT (20 μl, 5 mg/mL) was added to each well and the plate was incubated for 4 h. Then, the medium content was extracted from each well and 100 μL of DMSO was added. After the formazan crystals were completely dissolved, the absorbance of the solutions at 570/640 nm was measured by the microplate reader. Finally, the absorbance ratio of the treated wells was compared with the control group.

#### Anti-cancer activity study on breast cancer cell line (4T1)

In vitro treatment efficacy was evaluated on the breast cancer cell line (4T1). Cells (5 × 10^3^ cells/mL, 100 µl/well) were seeded in 96-well plate and incubated for 24 h. Cells were treated with Au@Alg-DA NPs for 12 h. Then it was exposed to X-ray and NIR. In the current assay, samples were treated in the absence and presence of Near-infrared (NIR) radiation (808 nm, 1 W/cm^2^, 5 min) and X-ray (4 Gy, 6 MV) with or without NPs. After an additional 24 h incubation, the viability of the cells was determined via MTT assay.

#### Intracellular reactive oxygen species (ROS) generation assay

To quantify intracellular ROS generation, we used 2′,7′-Dichlorofluorescin diacetate (DCFH-DA), a fluorescent dye that measures the production of hydroxyl, peroxyl, and other forms of ROS within cells. After uptake by cells, DCFH-DA is converted to a non-fluorescent molecule through deacetylation by cellular esterases and then oxidized by ROS to produce green fluorescence. ROS production induced by the designed NPs was assessed by 5 × 10^3^ 4T1 cells incubated overnight in a 96-well plate. Then they were treated with NPs for 12 h. There is 8 groups including culture medium (control group), X-ray, NIR, X-ray + NIR, Au@Alg-DA, Au@Alg-DA + X-ray, Au@Alg-DA + NIR and Au@Alg-DA + X-ray + NIR. To assess the generation of reactive oxygen species (ROS), the prepared groups underwent co-incubation for an additional hour. Subsequently, 100 μL of DCFH-DA, a fluorescent probe for ROS detection at a concentration of 10 μM, was added to each well. The plates were then incubated for another hour to allow for probe uptake. Samples related to radiotherapy and photothermal therapy were then exposed to X-rays (4 Gy and 6 MV) and NIR (808 nm, 1 W/cm^2^) and then visualized using fluorescence microscopy.

#### Calcein AM/PI staining

The Calcein AM/PI (propidium iodide) assay is a method for determining the number of live and dead cells using green and red fluorescence, respectively. In a 96-well plate, 5 × 10^3^ 4T1 cells were incubated for 24 h. Then, samples were loaded onto the plates for 12 h in 8 groups as culture medium (control group), X-ray, NIR, X-ray + NIR, Au@Alg-DA, Au@Alg-DA + X-ray, Au@Alg-DA + NIR and Au@Alg-DA + X-ray + NIR, and incubated for another 24 h. Finally, Calcein AM (3 μM) was added to each well and incubated for 30 min, followed by the addition of PI (4 μM) for 5 min to produce green (live) and red (dead) fluorescence images of 4T1 cells in the 8 groups mentioned above.

#### Colony formation assay

The colony formation assay is a widely used in vitro method to assess the ability of a single cell to form a colony, a group of cells derived from a single cell that can divide and develop into a visible mass. This assay is frequently used in cell biology and cancer research to evaluate the clonogenic potential of cells and to examine the effects of treatments such as chemotherapy or radiation on cell growth and survival. Specifically, 4T1 cells were plated in a 6-well plate at a density of 200 cells per well and incubated for 24 h in a cell culture medium. After treatment, cells were kept in the incubator for an additional 10 days. Then, they were washed with PBS and fixed with a mixture of methanol and acetic acid (3:1). After a 5-min incubation, cells were stained with 0.5% crystal violet in methanol. After 15 min, the contents of each well were washed with deionized water and the number of colonies were counted. The cell survival fraction was calculated using these formulas:

$${\text{Plating}}\;{\text{efficiency}} = \frac{Surviving\;colonies }{{seeded\;cells}} \times \, 100$$

$${\text{Surviving}}\;{\text{Fractions}}\left( \% \right) = \frac{Surviving\;colonies }{{seeded\;cells \times plating\;efficiency\;of\;control}} \times \, 100$$

### In vivo experiment

#### Evaluation of Au@Alg-DA in murine modal (Balb/c)

1 × 10^6^ 4T1 cells were injected subcutaneously into the right flank of Balb/C mice to generate a tumor. Once the tumor reached a volume of 280 mm^3^, the mice were randomly divided into 8 groups: Control group, X-ray, NIR, X-ray + NIR, Au@Alg-DA, Au@Alg-DA + X-ray, Au@Alg-DA + NIR and Au@Alg-DA + X-ray + NIR. The treatment plan included intraperitoneal injection of the assigned group 24 h prior, followed by exposure to single dose of X-ray and NIR three times. The mice were irradiated with X-ray (4 Gy, 6 MV) and high power LED (1 W/cm^2^, 2 min) and returned to the animal chamber. Tumor size and weight of the mice were measured over 21 days. The tumor volume was calculated using a mathematical formula.

$${\text{Tumor}}\;{\text{Volume}}\;\left( {{\text{mm}}} \right) = \frac{{\left( {Tumor\;length} \right) \times (Tumor\;width)^{2} }}{2} \times 100$$

#### Histology study

Tumor and organ tissues such as spleen, kidney, heart, and liver from control and Au@Alg-DA NPs + X-ray + NIR groups were harvested after 21 days and preserved in 10% formalin solution. The samples were then forwarded to a histology lab for histopathological examination.

### Statistical analysis

All quantitative data were declared as mean with standard deviation (mean ± SD) unless otherwise stated. Statistical analysis was depicted using GraphPad Prism 8 software.

### Ethics approval

All methods used in this study were conducted in accordance with relevant guidelines and regulations of the Ethics Committee of the Ahvaz Jundishapur University of Medical (Code: 99026551). The study is reported in accordance with ARRIVE guidelines.

## Results and discussion

### Synthesis and characterization

**Figure 1:**
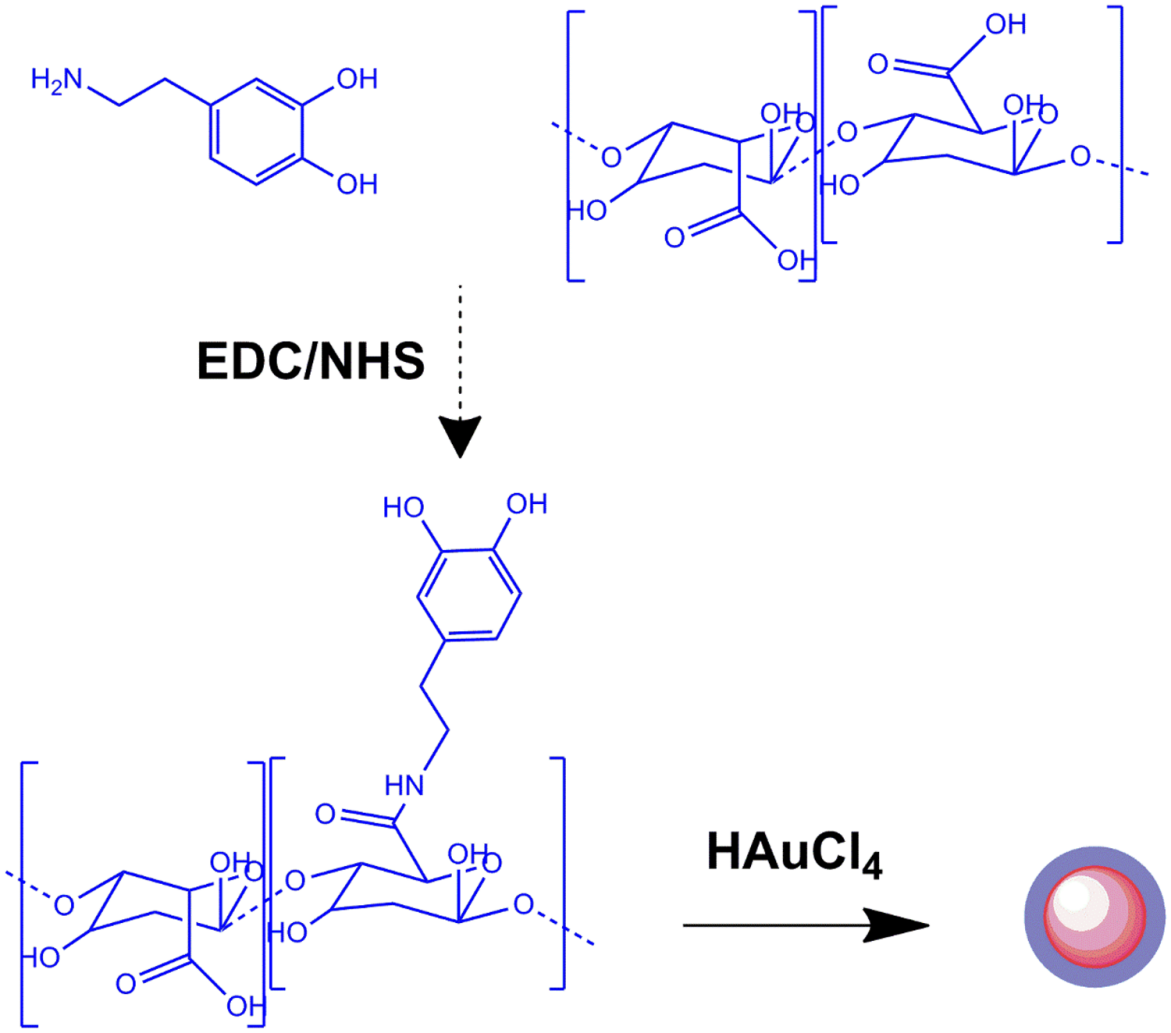
Schematic illustration of synthesis process of Au@Alg-DA NPs.

The literature has covered the production and characterization of Alg-DA polymer in depth^27,28^. As shown in Fig. 1, Alg-DA was formed utilizing EDC/NHS chemistry. A very unstable and hydrolyzable O-acylisourea intermediate was formed when EDC interacted with the carboxyl groups of Alg. The O-acylisourea intermediate could be made more stable by adding NHS, which would turn it into an amine-reactive NHS ester (succinimidyl ester) while allowing for efficient conjugation to primary amines. The final Alg-DA product was created when the amine-reactive ester reacted with the amine groups on DA. Our study expands on the existing methods for Alg-DA synthesis using EDC/NHS chemistry, which is aligned with previous research^29,30^.

**Figure 2:**
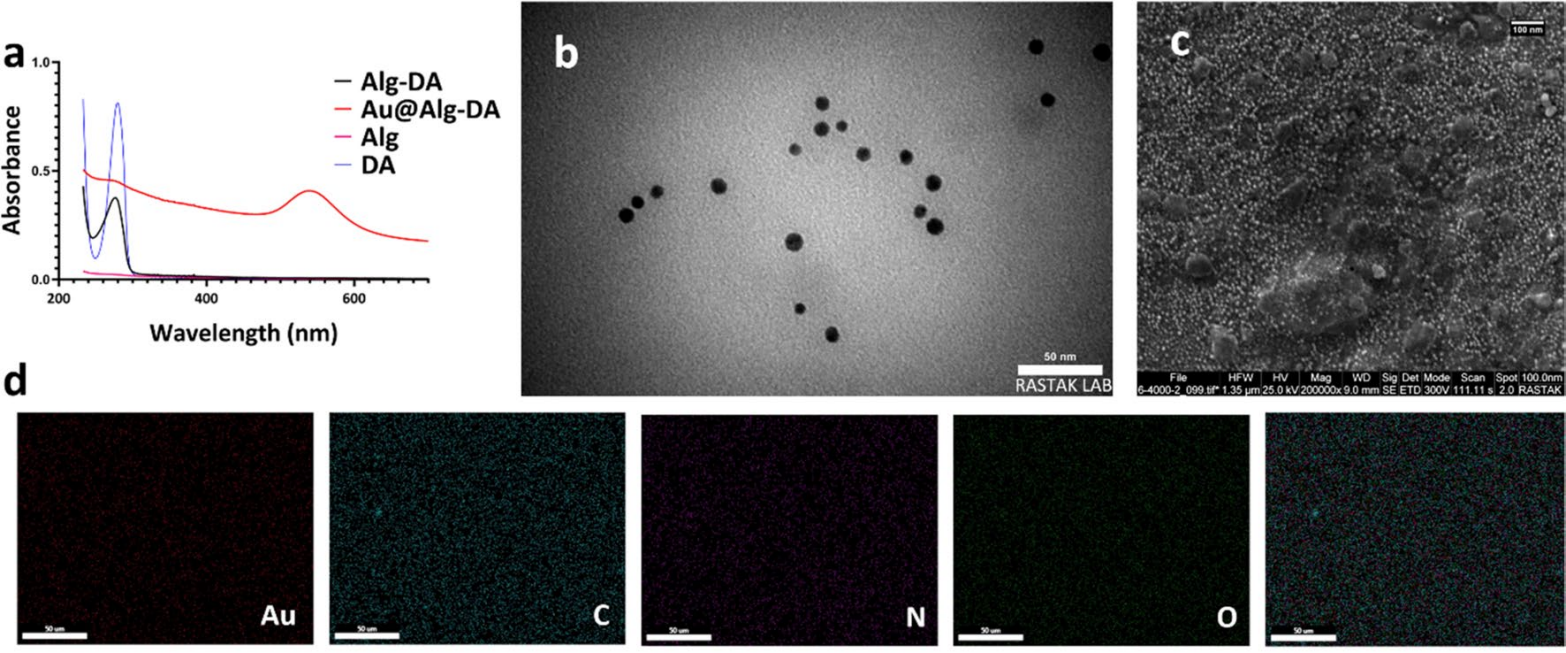
Characterization. (**a**) Uv–Vis spectra of DA, Alg, Alg-DA, and Au@Alg-DA; (**b**) TEM images of Au@Alg-DA NPs; (**c**) Fe-SEM images of Au@Alg-DA NPs; (**d**) SEM–EDS mapping of Au@Alg-DA NPs.

Figure 2a displays the results of monitoring the UV–Vis spectra of samples. There is no characteristic peak for Alg solution, only the absorbance slightly increases around 330 nm. However, UV–vis spectrum of DA shows an absorbance at 279.7 nm. Evidence of successful binding of DA to the Alg backbone came from the UV–Vis absorbance at ~ 280 nm in Alg-DA and DA samples. DA conjugation resulted in the appearance of a new peak at 276 nm, which was attributed to stretching of the DA catechol group^27,31^. In addition, Fig. S1 shows that DA was effectively conjugated onto Alg, as peaks between 6.5 and 7.0 ppm appeared in the ^1^H NMR spectra of the Alg-DA sample compared to Alg^32^. Binding of DA to the Alg backbone was further confirmed by methylene group peaks at 2.7 and 3.1 ppm^33^. The successfully synthesized Alg-DA polymer was then used as both a reducing and coating agent in the preparation of Au NPs. The color change of the solution to ruby red served as the earliest evidence for the successful synthesis of gold NPs (Au@Alg-DA) by the green and facile method. Au NPs show surface plasmon resonance (SPR) absorption extensively used to confirm their successful synthesis^34^. Figure 2a displays the UV–Vis spectrum of Au@Alg-DA. The SPR absorption peak of Au@Alg-DA NPs was centered at about 540 nm. The UV–Vis spectrum of Au@Alg-DA NPs not only shows the characteristic SPR peak of Au at 540 nm, but also show DA’s characteristic peak at ~ 270 nm. This peak of DA in the UV–Vis spectrum of Au@Alg-DA NPs shows a blue shift due to the interaction of Alg-DA with Au NPs. The SPR peak seen at 540 nm aligns with the typical range of gold nanoparticles, commonly reported as 520–570 nm^35^. Additionally, the blue shift of the DA peak observed post-conjugation is in line with findings from previous studies, which indicate an interaction between the biopolymer and the gold nanoparticles^36^.

TEM and FeSEM images in Fig. 2b,c reveal the spherical and monodisperse morphology of the synthesized Au@Alg-DA NPs. In the presence of Alg-DA, synthesis yields uniform and monodispersed NPs, while there is much aggregation in Au NPs obtained by neat Alg, without DA modification (data not shown). The SEM–EDS mappings in Fig. 2d confirmed the presence of Au, C, N, and O elements in the composition of NPs. The elements are uniformly distributed in the structure of NPs.

**Figure 3:**
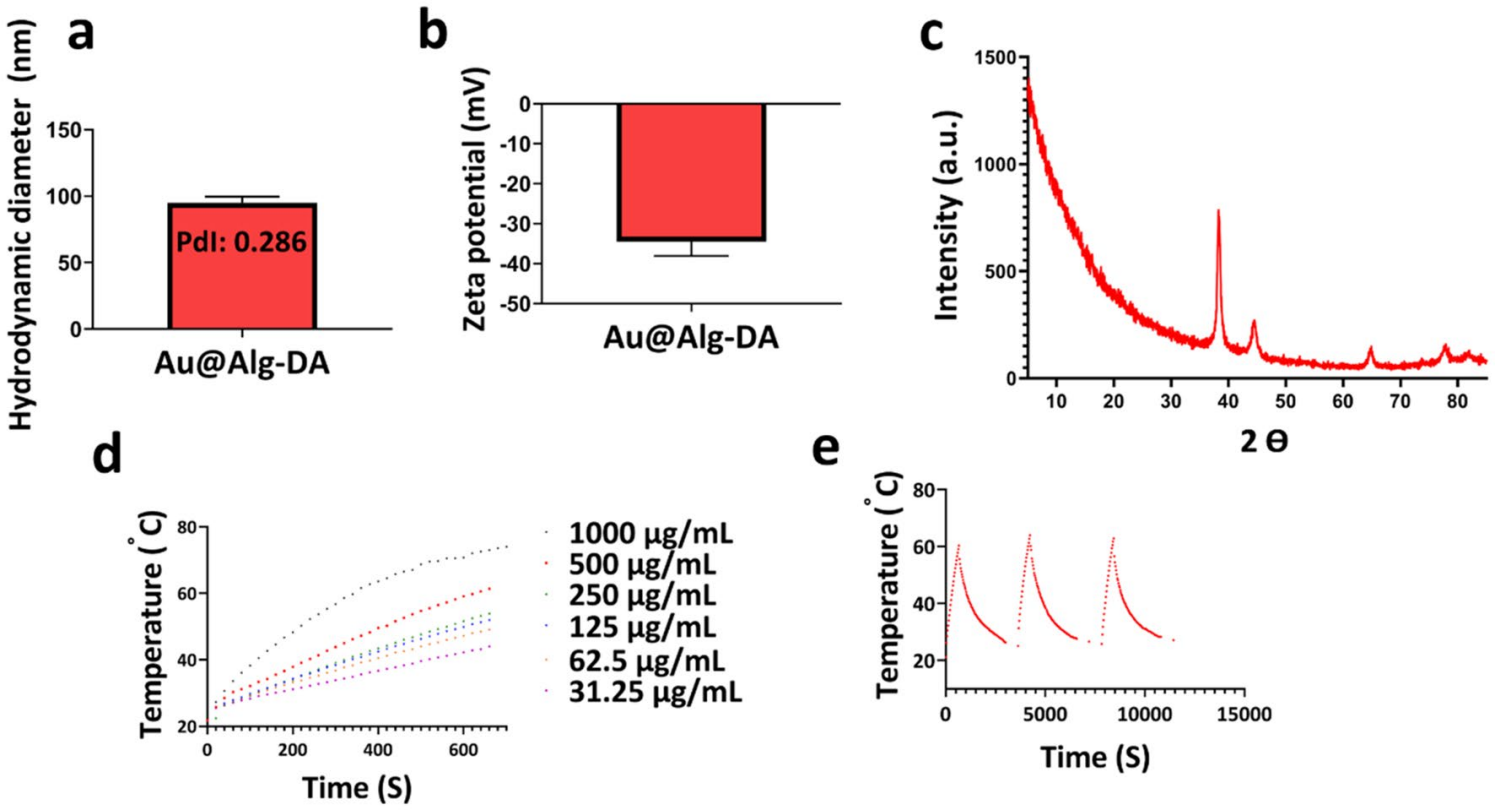
Characterization and Photothermal studies. (**a**) Hydrodynamic size of Au@Alg-DA NPs; (**b**) Zeta potential of Au@Alg-DA NPs; (**c**) XRD pattern of Au@Alg-DA NPs; (**d**)) The temperature change profiles of Au@Alg-DA NPs under the irradiation by 808 nm LED (1.0 W/cm^2^); (**e**) Temperature change of Au@Alg-DA NPs over three cycles of repeated irradiation.

The mean particle size obtained from TEM analysis was ca. 8.7 ± 1.3 nm. However the hydrodynamic size of the Au@Alg-DA NPs was found to be higher, about 95 nm, compared to their dry state (Fig. 3a). NPs have a negative surface charge of about − 34.5 mV (Fig. 3b). The hydrodynamic diameter of Au@Alg-DA NPs remained stable in deionized water for 30 days, as shown in Fig. S2. Our discovery of Au@Alg-DA nanoparticles, which are spherical and uniform, measures approximately 8.7 nm. This finding is consistent with earlier research that used biopolymer-based methods to synthesize gold nanoparticles^37,38^. However, other studies indicate that even smaller sizes can be achieved by modifying the biopolymer used and adjusting reaction conditions^39,40^.

The crystallinity of the as-prepared Au@Alg-DA NPs was studied by XRD, and the pattern was shown in Fig. 3c, where the characteristic (111), (200), (220), (311), and (222) lattice planes of metallic gold in the fcc phase (JCPDS # 04-0783) can be seen in the diffraction patterns at 38.2, 44.5, 64.9, 77.7, and 81.9°^41^. The XRD pattern confirms the formation of face-centered cubic (fcc) gold nanoparticles, which aligns with previous studies on biopolymer-synthesized gold nanoparticles^42^.

### Photothermal effects of Au@Alg-DA

As shown in Fig. 3d, after 320 s of LED exposure, the temperature of the aqueous Au@Alg-DA NPs solution (500 µg/mL) rose to 45 °C, while the temperature of the NPs-free sample changed little. When exposed to 808 nm LED radiation, Au@Alg-DA NPs showed concentration dependent continuous heating characteristic. The temperature curves did not change at all after three on/off cycles of the LED, indicating that the Au@Alg-DA NPs are very photothermally stable (Fig. 3e). Therefore, Au@Alg-DA NPs have shown a great potential for use in photothermal therapy. All in all, it was found that Au@Alg-DA NPs were successfully synthesized, had the expected structure, size and properties, and were stable enough to be used in further bioactivity tests. The heating behavior of the nanoparticles aligns with established principles of photothermal therapy, with higher concentrations of photothermal agents resulting in a greater rise in temperature when exposed to light^43^.

It is particularly encouraging that the Au@Alg-DA NPs maintain their heating efficiency after multiple on/off cycles. This suggests good photothermal stability, which is a crucial requirement for practical applications in photothermal therapy^44^.

### Cytotoxicity

**Figure 4:**
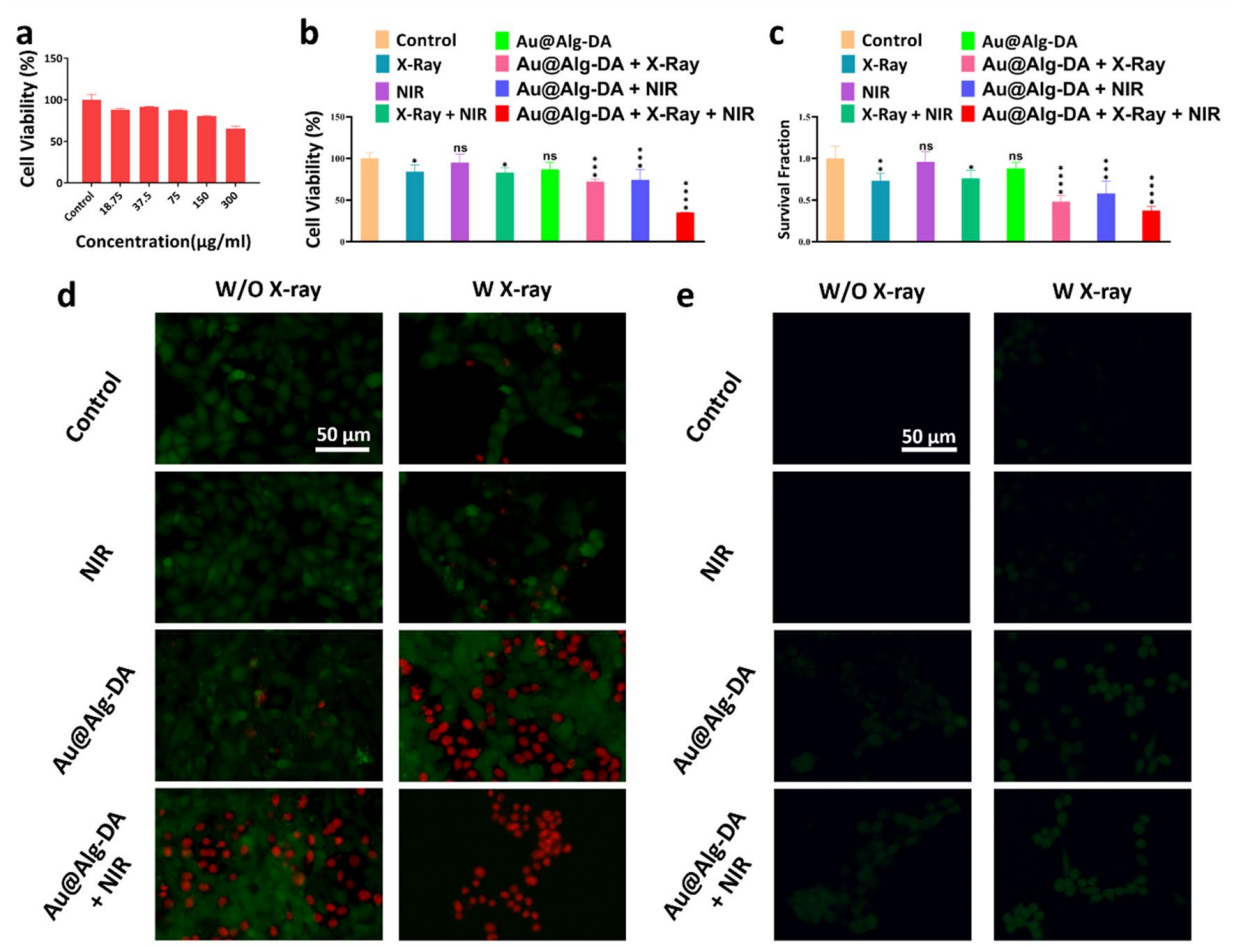
In vitro assays. (**a**) Cell viability result of HUVEC cells treated with different concentrations of Au@Alg-DA NPs; (**b**) Cell viability result of 4T1 cells treated with Au@Alg-DA NPs with and without irradiation; (**c**) Survival fraction of 4T1 cells treated with Au@Alg-DA NPs with and without irradiation; (**d**) Fluorescence microscopy images of the treated and co-stained 4T1 cancer cells. Calcein AM is used to stain the live cells. PI is used to stain the dead cells (e) Intra-cellular ROS generation assay after different treatments.

The biocompatibility of Au@Alg-DA NPs was evaluated at the cellular level by MTT assay. Upon incubation with HUVEC cells, no substantial cytotoxicity was detected in the presence of 18.75 μg/mL, 37.5 μg/mL, 75 μg/mL, and 1500 μg/mL Au@Alg-DA NPs, as cell viability remained above 80% in each group. This result, depicted in Fig. 4a, attests to the biocompatibility of Au@Alg-DA NPs.

The MTT assay was implemented to evaluate the potential anticancer effect of Au@Alg-DA NPs as depicted in Fig. 4b. The control group exhibited a complete cell viability as expected. 4T1 breast cancer cells did not change much in viability when exposed to X-ray (4 Gy and 6 MV) and NIR (1 W/cm^2^, 5 min) radiations (almost 84% and 95%, respectively). It is apparent that the bare X-ray group was more detrimental to cell viability compared to the bare NIR group. When Au@Alg-DA NPs were administrated in combination with X-ray or NIR individually, cell viability was decreased compared to the radiation-alone protocols, although the cell viability of these two groups was quite close. Favorably, our findings demonstrate that simultaneous application of Au@Alg-DA NPs, X-ray, and NIR resulted in a substantial decrease in cell viability (~ 35%), highlighting the potential of combination therapy approaches in cancer treatment.

Our findings show that Au@Alg-DA NPs are not significantly toxic to HUVEC cells at concentrations up to 150 μg/mL, indicating their promising biocompatibility. However, it's important to note that cytotoxicity can vary depending on the cell type and duration of exposure. The observation that X-ray exposure (4 Gy) has a more pronounced cytotoxic effect on 4T1 cells compared to NIR radiation (1 W/cm^2^, 5 min) aligns with existing knowledge. X-rays are generally known to be more cytotoxic due to their higher energy and ability to cause greater damage to DNA^45^. The main finding of our study is that combining Au@Alg-DA NPs with both X-ray and NIR irradiation significantly reduces 4T1 cell viability (approximately 35%) compared to treatment with either radiation alone. This is an encouraging result that supports the concept of combination therapy in cancer treatment, where multiple modalities can work together to enhance treatment efficacy and potentially reduce side effects. The heating behavior of the nanoparticles aligns with established principles of photothermal therapy, with higher concentrations of photothermal agents leading to a greater rise in temperature when exposed to light^46^. It is particularly encouraging that the Au@Alg-DA NPs maintain their heating efficiency after multiple on/off cycles. This suggests good photothermal stability, which is a crucial requirement for practical applications in photothermal therapy^47^.

### Colony formation assay

To further assess the anti-cancer efficacy of the treatment group, a colony assay was also performed. As depicted in Fig. 4c, the control group exhibited a complete survival fraction. X-ray (4 Gy and 6 MV) and NIR (1 W/cm^2^, 5 min) radiations did not appear to significantly affect the survival rate of 4T1 breast cancer cells. The bare X-ray group, on the other hand, was found to have a greater adverse effect on cell growth compared to the NIR group, which is quite concordant with cytotoxicity results. Our data showed that administration of Au@Alg-DA NPs in combination with X-ray or NIR decreased colony formation significantly. The reduction was greater in the case of X-ray + NPs treatment compared to X-ray-alone and NPs-alone protocols. The triple combination treatment (Au@Alg-DA NPs + X-ray + NIR) further reduced colony formation and offered the lowest rate (~ 0.37). Our results indicate that the simultaneous application of Au@Alg-DA NPs, X-ray, and NIR resulted in a marked decrease in the survival fraction of cancer cells, signifying the successful anti-cancer effect of our design.

The results of our colony formation test support the results of the MTT test and strengthen the potential of Au@Alg-DA NPs for combination therapy in the treatment of breast cancer. The observation that X-ray irradiation (4 Gy) has a minimal effect on colony formation of 4T1 cells, while NIR irradiation has an even weaker effect, is consistent with some studies. However, other studies report a more pronounced effect of X-rays on colony formation depending on the specific cell line, radiation dose and other experimental conditions^48^. Furthermore, the combination of Au@Alg-DA NPs with X-ray and NIR irradiation significantly reduced colony formation compared to treatment with either radiation alone. This is a strong result that supports the potential of this approach to improve therapeutic efficacy. Similar results have been reported in other studies investigating combination therapy with gold nanoparticles for radiosensitization or photothermal therapy of cancer cells^49,50^.

### Calcein-AM/PI cell staining assay

The Calcein-AM/PI cell staining assay was utilized to evaluate the ability of papered samples to induce cell death. This method involves the use of Calcein-AM and PI reagents to differentiate between live and dead cells. After treatment with various agents, 4T1 cells were stained with the aforementioned dyes and visualized under a microscope (as depicted in Fig. 4d). Calcein-AM, acting as a green fluorescent dye, was utilized to label live cells, whereas PI, a red fluorescent dye, was used to identify dead cells^19^. The increased rate of cell death is represented by the increased intensity of red colored regions, while the presence of viable cells is indicated by green spots.

In the control groups, the predominant fluorescence was green as depicted in Fig. 4d. Individual exposure to X-rays or NIR irradiation had minimal effects on cell death, as evidenced by the limited number of red spots found in 4T1 cancer cells. Treatment of cells with Au@Alg-DA NPs resulted in a higher rate of green fluorescence along with a modest presence of red fluorescence. Co-administration of X-rays and NIR with NPs resulted in an elevated incidence of cell death, as evidenced by the increased red fluorescence intensity. The most notable increase in red fluorescence and the accompanying decrease in green fluorescence were observed in cells treated with Au@Alg-DA NPs in combination with both X-rays and NIR irradiation, signifying the potent anticancer properties of these NPs. This remarkable decrease in the viability of cancer cells can be attributed to the combinational effect of ROS generation by X-ray exposure when high-Z metals such as gold are used and heat release under NIR^51^.

The results of the calcein-AM/PI staining test visually confirm the results of the MTT and colony formation test, further supporting the potential of Au@Alg-DA NPs for combination therapy in breast cancer. The use of calcein-AM and PI to differentiate between live and dead cells is a well-established technique^52^. Our observation that the control cells show predominantly green fluorescence (live cells) is in line with expectations. The finding that individual exposure to X-ray or NIR radiation has minimal effect on cell death observed by limited red fluorescence is consistent with some studies, especially at lower doses. The increased red fluorescence (dead cells) when Au@Alg-DA NPs are co-administered with X-ray or NIR radiation is consistent with your previous findings and supports the concept of combination therapy. Similar results are reported in other studies investigating the use of gold nanoparticles to enhance the cytotoxicity of radiation or light irradiation on cancer cells^53^. The combination of gold-alginate nanoparticles, X-ray, and near-infrared light showed the most significant increase in red fluorescence and decrease in green fluorescence. This provides strong visual evidence for the enhanced anti-cancer effect of this combined approach^54^.

### ROS generation assay

The utilization of X-rays as a means of cancer treatment is augmented by the induction of DNA damage through radiation. Measurement of intracellular ROS generation was made using the DCFH-DA fluorescent probe, which emits a bright green fluorescence when oxidized^55^. As shown in Fig. 4e, the experimental control group not exposed to X-ray or NIR irradiation exhibited a lack of green fluorescence, indicating an absence of ROS production. However, cells treated solely with X-rays irradiation demonstrated a slight green fluorescence. As anticipated, cells treated with Au@Alg-DA NPs without applying X-rays or NIR exhibited limited green fluorescence. However, the green fluorescence intensity in cells treated with both Au@Alg-DA NPs + X-rays was notably higher than that of cells treated with Au@Alg-DA NPs alone. Interestingly, cells treated with the combined application of X-rays, NIR and Au@Alg-DA NPs displayed the highest level of green fluorescence. Consequently, the synergistic effect of X-rays and NIR in the presence of Au@Alg-DA NPs significantly augments ROS production. It is well known that X-ray irradiation alone leads to a slight increase in ROS generation. X-rays can interact with water molecules within cells, resulting in the production of reactive oxygen species^56^. Furthermore, our observation that Au@Alg-DA NPs alone have minimal impact on ROS production is consistent with previous studies, especially when used in low concentrations^57^. The most significant finding of our study is that the combination of Au@Alg-DA NPs with X-ray or NIR irradiation significantly enhances ROS generation compared to either treatment alone. This result is particularly interesting and supports the potential of this approach in improving the effectiveness of cancer therapy. Similar findings have been reported in other studies, suggesting that gold nanoparticles can function as radiosensitizers by promoting ROS generation upon exposure to X-rays^53,58^.

### In vivo anticancer study

**Figure 5:**
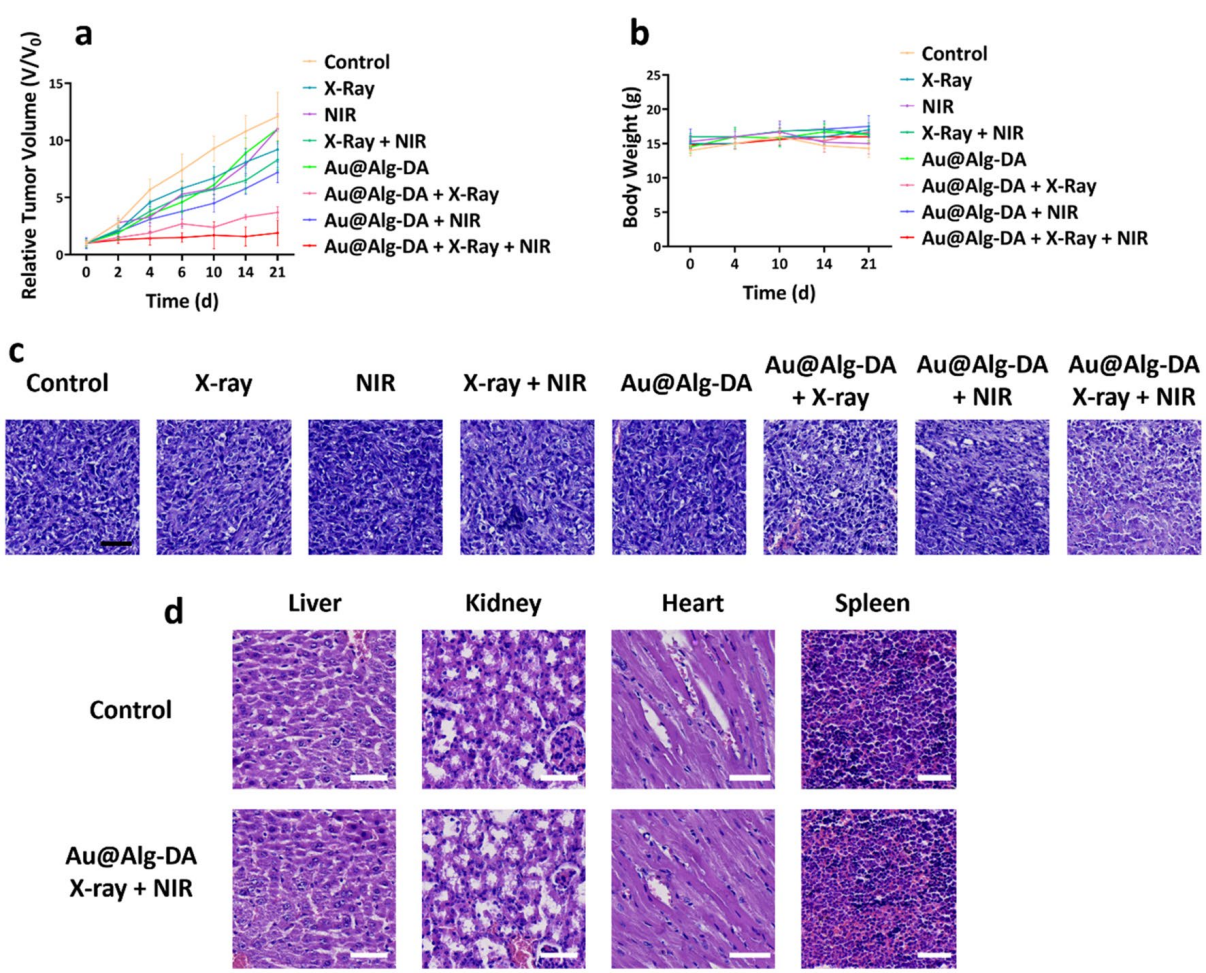
In vivo anticancer ability. (**a**) The tumor growth profile; (**b**) body weight of tumor-bearing mice after treatment with different plans; (**c**) H&E-stained tumor sections of mice treated with different treatment plans; and (**d**) H&E-stained tissue sections of heart, liver, spleen and kidney from mice treated with saline or Au@Alg-DA NPs + X-ray + NIR.

Based on the in vitro assay findings, we proceeded to evaluate the anti-cancer effects of the experimental groups in Balb/C mice. The radio-enhancing capacities were analyzed in murine model. After obtaining a mean tumor volume of 280 mm^3^, the mice were randomly allotted into 8 groups and the treatment was initiated. Mice were subjected to X-rays (4 Gy and 6 MV) and NIR irradiation (1 W/cm^2^, 2 min) 24 h after NPs injection. The results of tumor size reduction following intraperitoneal administration are displayed in Fig. 5a. The control group did not demonstrate any tumor growth inhibition effect, and the utilization of Au@Alg-DA NPs alone was also ineffective in curbing tumor growth. Administration of X-rays or NIR radiation alone showed a marginal improvement in anticancer activity, however, the combination of intravenous/intraperitoneal injection of NPs with X-rays or NIR resulted in a higher tumor growth inhibition. The group treated with Au@Alg-DA NPs + X-rays displayed a greater tumor growth inhibitory effect compared to that observed with Au@Alg-DA NPs + NIR. The highest tumor growth inhibitory effect was observed when mice were exposed to combined treatment with X-rays and NIR in the presence of Au@Alg-DA NPs. Additionally, the body weight of the mice was meticulously monitored throughout the experimental period (Fig. 5b) and no significant changes were observed in all experimental groups, supporting the safety and efficacy of Au@Alg-DA NPs as a radiosensitizer and photothermal agent in cancer treatment The effectiveness of in vivo treatment was evaluated using hematoxylin and eosin (H&E) staining on both healthy and tumor tissue to determine whether the treatment causes any damage in tumor and has any adverse effects on healthy organs. As can be seen from Fig. 5c,d, the biocompatibility of NPs for use in vivo breast cancer treatment can be highlighted by the fact that a comprehensive evaluation of major organs, such as kidneys, spleen, liver, and heart, did not reveal any obvious tissue damage.

The cellular state of the tumors in the control group did not differ significantly from that of the groups treated with X-rays, NIR, and Au@Alg-DA NPs. However, tumor tissue was somewhat damaged when Au@Alg-DA NPs were used in combination with X-rays or NIR. Large shadow regions and necrotic-shaped cells were seen in the groups received treatment with Au@Alg-DA NPs together with X-rays and NIR, demonstrating effective removal of tumor cells. The observation that neither Au@Alg-DA NPs alone nor X-ray or NIR irradiation alone significantly inhibits tumor growth aligns with some studies, highlighting the limitations of these individual approaches^59^. The finding that the combination of Au@Alg-DA NPs with X-ray or NIR irradiation results in a more pronounced tumor growth inhibition effect compared to either treatment alone is a key result. This is consistent with studies exploring the use of gold nanoparticles for radiosensitization or photothermal therapy in vivo^49,60^. The most significant tumor growth inhibition observed with the triple combination (Au@Alg-DA NPs + X-ray + NIR) treatment strengthens the overall conclusion. This is encouraging evidence for the potential of this approach in vivo. The finding that no significant changes in body weight are observed across all groups suggests good biocompatibility of Au@Alg-DA NPs in vivo, which is an important consideration for cancer therapy. Similar results are reported in other studies using biopolymer-coated gold nanoparticles^61^. The use of hematoxylin and eosin (H&E) staining for evaluating tissue damage is a standard technique^62^. The observation that no obvious tissue damage is detected in major organs (kidney, spleen, liver, heart) of treated mice compared to controls supports the biocompatibility of Au@Alg-DA NPs in vivo. The finding that tumor tissue from the triple combination group displays the most significant damage (shadow regions, necrotic cells) compared to other groups aligns with the in vitro results and suggests effective tumor cell removal. 

## Conclusions

We introduced a green and facile synthesis of gold AuNPs using Alg-DA as a reducing and stabilizing agent. The presence of DA in the side groups of Alg allows for a safe and efficient AuNP synthesis and improved biocompatibility, as well as provides more uniform Au NPs in shape and size. Au@Alg-DA NPs not only generated ROS within cancer cells under X-ray irradiation, but were also able to increase the temperature of cells when exposed to NIR. The ROS generation assay showed a significant ROS activity increase in cells treated with Au@Alg-DA NPs in combination with X-ray and NIR. Mice treated with all three of the X-rays, NIR irradiation, and intraperitoneal injection of Au@Alg-DA NPs experienced a higher tumor growth inhibition compared to those administered with NPs, X-rays or NIR alone. The cellular state of tumors in the groups treated with X-rays, NIR, or Au@Alg-DA NPs did not differ significantly, but tumor tissue was somewhat damaged when Au@Alg-DA NPs were used in combination with both X-rays and NIR, indicating the efficient elimination of cancer cells. Our data support the use of a biocompatible NP system that acts as both a radiosensitizer for effective radiotherapy and an efficient photothermal conversion agent for photothermal therapy. The combination of strong radiosensitizing effect and NIR absorption makes these Au@Alg-DA NPs ideally suited for combined treatment of cancer.

### Supplementary Information

Supplementary Figures.

## Data Availability

The raw/processed data required to reproduce these findings cannot be shared at this time as the data also forms part of an ongoing study.
