# Supplementary material for: Photothermal and radiotherapy with alginate-coated gold nanoparticles for breast cancer treatment
Source: Sci Rep. 2024 Jun 10;14:13299. doi: 10.1038/s41598-024-60396-w (PMC11164878; doi:10.1038/s41598-024-60396-w)
Supplement: Supplementary file 1 — Supplementary Figures. [file 41598_2024_60396_MOESM1_ESM.docx]

**Photothermal and Radiotherapy with Alginate-Coated Gold Nanoparticles for Breast Cancer Treatment**

Mohammadreza Ghaffarlou^1^, Hamid Rashidzadeh^2^, Ali Mohammadi^2^, Navid Mousazadeh^2^, Murat Barsbay^1^, Ali Sharafi^2^, Mahmoud Gharbavi^3,4*^, Hossein Danafar^2*^, Siamak Javani^5,6*^

1. Hacettepe University, Department of Chemistry, Beytepe, Ankara 06800, Turkey.
2. Zanjan Pharmaceutical Biotechnology Research Center, Zanjan University of Medical Sciences, Zanjan, Iran.
3. Nanotechnology Research Center, Ahvaz Jundishapur University of Medical Sciences, Ahvaz, Iran.
4. Pain Research Center, Imam Khomeini Hospital Clinical Research Development Unit, Ahvaz Jundishapur University of Medical Sciences, Ahvaz, Iran.
5. Medical Cellular and Molecular Research Center, Golestan University of Medical Sciences, Gorgan, Iran.
6. School of Advanced Technologies in Medicine, Golestan University of Medical Sciences, Gorgan, Iran.

*Corresponding Authors:

Mahmoud Gharbavi; Email: [gharbavi1981@gmail.com](mailto:gharbavi1981@gmail.com)

Siamak Javani; Email: [siamackjavani@yahoo.com](mailto:siamackjavani@yahoo.com)

Hossein Danafar; Email: [danafar@zums.ac.ir](mailto:danafar@zums.ac.ir)

**Number of pages: 3**

**Number of figures: 2**

**Number of tables: 0**

## Supporting figures


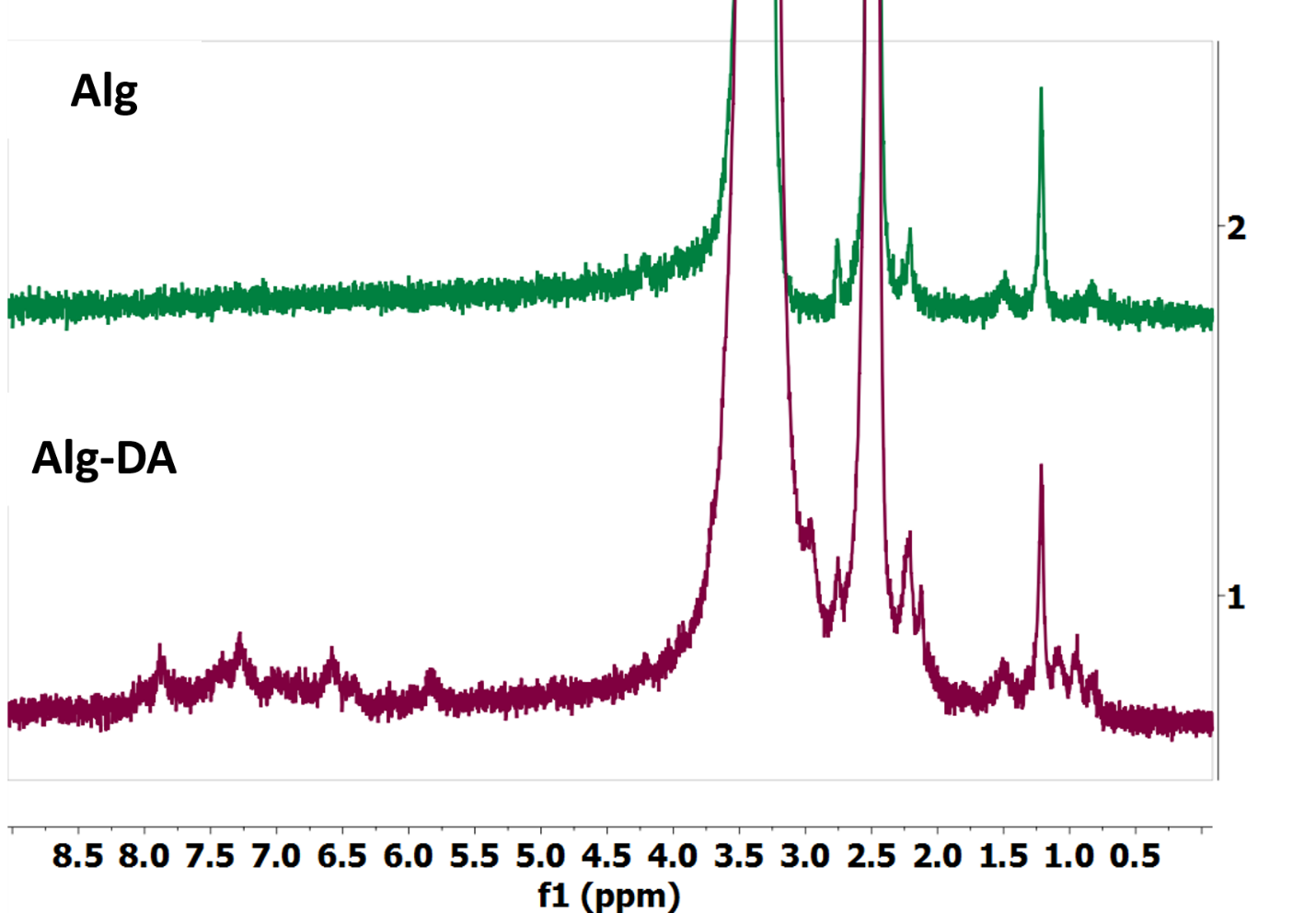


**Fig. S1.** ^1^HNMR spectrum of Alg-DA (1) and Alg (2).


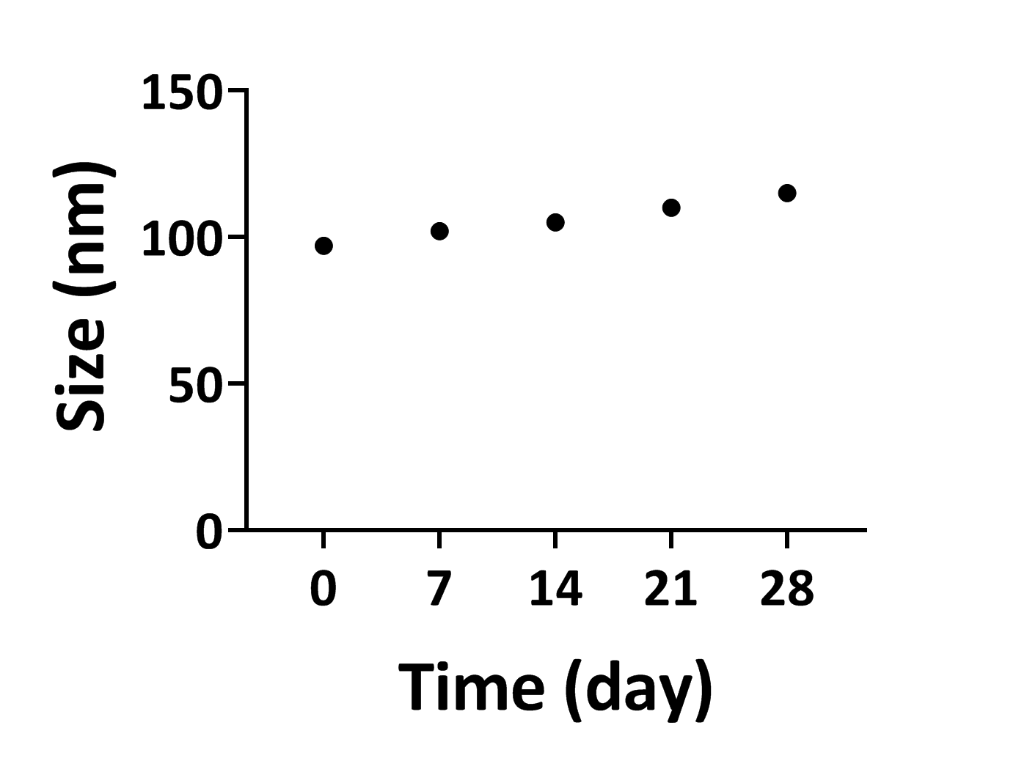


**Fig. S2.** Hydrodynamic size of Au@Alg-DA NPs over 4 weeks.
